# The gut microbiota and inflammatory bowel disease

**DOI:** 10.1007/s00281-014-0454-4

**Published:** 2014-11-25

**Authors:** Katsuyoshi Matsuoka, Takanori Kanai

**Affiliations:** Division of Gastroenterology and Hepatology, Department of Internal Medicine, Keio University School of Medicine, 35 Shinano-machi, Shinjuku, Tokyo 160-8582 Japan

**Keywords:** Inflammatory bowel disease, Ulcerative colitis, Crohn’s disease, Dysbiosis

## Abstract

Inflammatory bowel disease (IBD) is a chronic and relapsing inflammatory disorder of the gut. Although the precise cause of IBD remains unknown, the most accepted hypothesis of IBD pathogenesis to date is that an aberrant immune response against the gut microbiota is triggered by environmental factors in a genetically susceptible host. The advancement of next-generation sequencing technology has enabled identification of various alterations of the gut microbiota composition in IBD. While some results related to dysbiosis in IBD are different between studies owing to variations of sample type, method of investigation, patient profiles, and medication, the most consistent observation in IBD is reduced bacterial diversity, a decrease of Firmicutes, and an increase of Proteobacteria. It has not yet been established how dysbiosis contributes to intestinal inflammation. Many of the known IBD susceptibility genes are associated with recognition and processing of bacteria, which is consistent with a role of the gut microbiota in the pathogenesis of IBD. A number of trials have shown that therapies correcting dysbiosis, including fecal microbiota transplantation and probiotics, are promising in IBD.

## Introduction

Inflammatory bowel disease (IBD) is a disorder characterized by chronic and relapsing intestinal inflammation and is mainly defined as either ulcerative colitis (UC) or Crohn’s disease (CD). Although the cause of IBD remains unknown, genetic background is considered to be involved in the pathophysiology of IBD because a number of disease susceptibility genes have been identified. The rapid increase in the incidence of IBD, however, cannot be explained by genetic factors alone, and environmental factors must also be essential to its development.

The involvement of the gut microbiota in the pathophysiology of IBD has recently been highlighted. Several lines of evidence suggest an essential role of the gut microbiota in intestinal inflammation. (1) In murine models of IBD such as IL-10-deficient mice and the CD45Rb^high^ transfer model, where transferred naïve helper T cells cause microbiota-dependent intestinal inflammation in immune-deficient recipients such as Rag2^−/−^ mice, germ-free animals do not develop colitis. (2) Diversion of the fecal stream ameliorates intestinal inflammation in CD [1]. (3) Antibiotics are, to a certain degree, effective for the treatment of IBD [2]. (4) Antibiotics such as metronidazole and ciprofloxacin are also effective for anal lesions and prevention of postoperative recurrence in CD [3]. (5) Many of the reported IBD susceptibility genes are associated with recognition and processing of microbes [4].

Given these observations, the most accepted hypothesis of IBD pathogenesis to date is that an aberrant immune response against the gut microbiota is triggered by environmental factors in a genetically susceptible host. In this review, we will discuss abnormalities of the gut microbiota observed in IBD, their contribution to the pathogenesis of IBD, and related therapeutic applications.

## Alteration of the gut microbiota in IBD

### Dysbiosis

The host provides a nutrient-rich environment and residence for the gut bacteria, and in turn, they contribute to the host by producing short-chain fatty acids and essential vitamins. This mutual relationship between the host and the gut bacteria is called symbiosis. Recent advancement of next-generation sequencing techniques has enabled culture-independent analysis of the gut microbiota, revealing that an altered balance of the gut microbiota constituents, rather than specific pathogens, is involved in the pathophysiology of several diseases. This shift in the balance of the gut microbiota is referred to as dysbiosis.

More than 90 % of the human gut microbiota is composed of four major phyla. The Firmicutes (49–76 %) and Bacteroidetes (16–23 %) phyla dominate, followed to a much less extent by the Proteobacteria and Actinobacteria phyla [5, 6]. The Firmicutes phylum is mainly composed of the *Clostridium* XIV and IV groups.

Various alterations of the gut microbiota have been reported in IBD patients (Table 1). Most studies have shown reduced diversity of the gut microbiota in IBD patients [6–9]. The most consistent observations of altered composition of the gut microbiota in IBD patients are a reduction in Firmicutes and an increase in Proteobacteria [6, 7, 10–12]. The reduced diversity of the gut microbiota observed in IBD patients is largely due to a decline in the diversity of Firmicutes. Among Firmicutes, a decrease in the *Clostridium leptum* groups, especially *Faecalibacterium prausnitzii*, has been reported in many studies [7, 13, 14]. Results related to Enterobacteriaceae, *Bacteroides*, *Bifidobacteria* species, *Lactobacillus* species, and *Escherichia coli* are not consistent among studies [15, 16]. Various factors may explain the between-study discrepancies: (1) sample source (biopsy or stool), (2) sampling location (inflammatory or noninflammatory sites), (3) disease activity (active or quiescent), (4) medication, (5) diet, (6) age, (7) smoking, and (8) methods used to analyze the microbiota.

**Table 1:** Metagenomic analysis of the gut microbiota in inflammatory bowel disease

Sample source	Sample no.			Diversity	Bacterial no.	Firmicutes	Bacteroidetes	Actinobacteria	Proteobacteria	Ref.
	CD	UC	HC							
Stool	6	–	6	↓ in CD		↓ in CD	→ in IBD			[7]
Biopsy	6	5	5			↓ in CD	↑ in CD	↑ in IBD	↑ in CD	[10]
Surgical tissue	35	55	34		↓ in IBD	↓ Lachnospiraceae	↓ in IBD	↑ Bifidobacteriaceae in cCD	↑ in IBD	[6]
						↑ *Bacillus*		↑ Coriobacteriaceae in cCD		
Stool	29	16	35	↓ in CD	↓ in CD	↑ in cCD, ↓ in iCD			↑ Enterobacteriaceae in CD	[8]
						↑ Ruminococcaceae in cCD				
						↓ Ruminococcaceae in iCD				
Biopsy	6	6	5	↓ in IBD	↓ in CD	↓ in IBD	↑ in IBD		↑ Enterobacteriaceae in CD	[24]
						→ *F. prausnitzii* in IBD				
Biopsy	121	75	27			↓ in CD		↑ in IBD	↑ Enterobacteriaceae in CD	[49]
Stool										
Endoscopic lavage	16	16	32	↓ in IBD		↓ in IBD			↑ in IBD	[9]
						↓ *F. prausnitzii* in IBD				
Stool	21	34	21		→	↓ *C. coccoides* and *C. leptum* in IBD		↑ *Bifidobacterium* in UC	↑ *E. coli* in CD	[14]
						↑ *Lactobacillus* in CD				
						↓*F. prausnitzii* in IBD				
Biopsy	29	15	21		↓ in IBD	↓ *C. coccoides* in CD	↑ in IBD	↓ Bifidobacteriaceae in CD	↑ *E. coli* in IBD	[14]
						↓ *C. leptum* in IBD				
						↑ *Lactobacillus* in CD				
						↓ *F. prausnitzii* in IBD				
Biopsy and stool	447	–	221	↓ in CD		↓ Clostridiales in CD	↓ in CD		↑ Enterobacteriaceae in CD	[20]

*CD* Crohn’s disease, *UC* ulcerative colitis, *IBD* inflammatory bowel disease, *iCD* ileal CD, *cCD* colonic CD

While the gut microbiota in healthy subjects shows little temporal change, the gut microbiota in IBD patients is unstable. The composition of the gut microbiota differs between active and quiescent stages. Furthermore, a study that longitudinally examined the gut microbiota in IBD patients for a year demonstrated that the gut microbiota was unstable even in UC patients in remission [17]. Before relapse of UC, normal anaerobic bacteria such as *Bacteroides*, *Escherichia*, *Eubacterium*, *Lactobacillus*, and *Ruminococcus* are decreased and the diversity of the gut microbiota is also reduced [18]. In CD patients, dysbiosis is observed even in patients with remission. Medication also affects the composition of the gut microbiota. Mesalazine, for example, reduces the total bacterial number to almost half [19]. Bowel rest, which is a treatment option in CD, changes the composition of the gut microbiota. Antibiotics dramatically amplify the dysbiosis of CD [20].

The distribution of the gut microbiota should be taken into account when interpreting dysbiosis. For example, the composition of the microbiota is significantly different between fecal and mucosal samples [5, 21]. Mucosal samples are reported to be superior in order to detect dysbiosis [20]. The mucosa-associated microbiota is increased in IBD compared with healthy subjects [22, 23]. It is tempting to speculate that the mucosa-associated microbiota is physiologically more important in IBD than luminal microbiota because of the close contact of mucosa-associated bacteria with the intestinal surface. It has also been reported that the gut microbiota is different in the same individual between inflammatory and noninflammatory sites [24]. The dysbiosis observed in noninflammatory sites may be more representative of a causative composition because the dysbiosis observed in inflammatory sites may be affected by inflammation.

It remains controversial whether dysbiosis is a cause or consequence of intestinal inflammation in IBD. Comparison of the gut microbiota composition of IBD patients with that of their unaffected relatives, who are likely to share genetic and environmental background, is useful to provide evidence relevant to this fundamental question. Compositional change of the gut microbiota was not consistent between UC patients and their unaffected twins [25]. In contrast, a decrease in *F. prausnitzii* was reported to be observed in both UC patients and their first-grade relatives [26]. Unaffected relatives of CD patients also had dysbiosis, although it was different from the dysbiosis observed in CD patients [27]. Furthermore, it was reported that the genetic status of *NOD2* and *ATG18L* genes, which are two major CD susceptibility genes, was associated with alteration of the gut microbiota [28]. These results suggest that dysbiosis is caused by genetic and environmental factors, rather than being a consequence of inflammation.

There have been attempts to utilize dysbiosis as a diagnostic tool or biomarker [9]. To date, there are no microbial constituents specific to UC or CD, because interindividual variations are much larger than inter-disease differences [6]. Several studies have suggested the possibility of using the gut microbiota as a biomarker. Firmicutes, for example, was increased in UC patients who responded to mesalazine [19]. The relapse rate was lower in postoperative CD patients who had a similar composition of gut microbiota to healthy controls than in those with dysbiosis [29]. In the largest CD microbiota cohort so far, comparing 447 newly diagnosed pediatric CD patients with 221 healthy controls, Gevers et al. proposed a “dysbiosis index,” which was shown to be associated with clinical disease severity assessed using the Pediatric Crohn’s Disease Index [20]. They also reported that profiles of the gut microbiota were able to be utilized as a diagnostic marker of CD and were also useful to predict the severity at 6 months. This report encourages further efforts to use gut microbial profiles as a diagnostic tool or biomarker for disease activity, prognosis, and response to treatment.

### Specific bacteria associated with IBD

There have been no specific pathogens yet identified that fulfill Koch’s postulates. There are, however, several specific bacteria that are associated with IBD. *Mycobacterium avium* subspecies *paratuberculosis* (MAP) causes chronic granulomatous ileitis (Johne’s disease) in cattle and sheep, which shares some pathological features with CD. In addition, because MAP has been found in commercial milk, it is suspected as a causative pathogen of CD. MAP was detected in CD patients using mucosal PCR, and the positive rates of serum anti-MAP antibody were higher in CD patients than in healthy controls or UC patients [30]. However, a clinical trial of a 2-year administration of antituberculosis drugs to CD patients showed no efficacy [31]. Adhesive-invasive *E. coli* (AIEC), which can adhere to and invade the intestinal epithelial cells, colonize the ileal mucosa of CD [32]. AIEC also replicate in macrophages and stimulate TNFα production from macrophages. It was observed that *Fusobacterium varium* attaches to inflamed regions in UC and invades the mucosa at ulcers [33]. Serum titers for anti-*F. varium* antibodies were higher in UC patients compared with healthy controls [34]. *F. varium* produces butyrate, and rectal administration of butyrate has been shown to cause mucosal damage in mice [35]. The pathological consequence of butyrate production by *F. varium*, however, needs to be examined more extensively because butyrate has diverse effects on the intestinal homeostasis including Treg induction in the gut and energy supply to the intestinal epithelial cells. The combination therapy of amoxicillin, tetracycline, and metronidazole (to which *F. varium* is sensitive) for 2 weeks showed efficacy in active UC patients, suggesting the possible pathogenic role of *F. varium* [36].

### The role of the gut microbiota in IBD revealed by susceptibility genes

The association of IBD susceptibility genes with bacteria has recently been highlighted. The development of the genome-wide association study has contributed greatly to the identification of more than 160 IBD susceptibility genes to date [4]. The physiological functions of these genes are categorized into several groups relating to (1) acquired immunity (*IL23R*, *IL12B*, *JAK2*, *STAT3*), (2) bacterial recognition and processing (*NOD2/CARD15*), (3) autophagy (*ATG16L*, *IRGM*, *ATG5*), and (4) mucosal barrier (*ECM1*, *CDH1*, *LAMB1*) [37]. Many of the CD susceptibility genes are associated with bacterial recognition and processing, and many of the UC susceptibility genes are related to mucosal barrier function, suggesting that impaired handling of bacteria or disruption of the mucosal barrier function leads to breakdown of tolerance against the commensal bacterial in the gut in CD and UC, respectively.

*NOD2/CARD15* was the first reported CD susceptible gene and shows the strongest association with CD. The function of the NOD2 protein has been extensively studied. NOD2 is an intracellular receptor for muramyl dipeptide (MDP), a component of the cell wall of gram-positive bacteria, and is expressed in intestinal epithelial cells and monocytes/macrophages. While *Nod2*-deficient mice do not develop spontaneous colitis, the bacterial load in the gut is increased in these mice. CD patients with *NOD2* mutations demonstrate diminished production of antimicrobial peptides (AMPs) from Paneth cells [38] as well as reduced production of the anti-inflammatory cytokine IL-10 from peripheral mononuclear cells [39]. NOD2 stimulation with MDP induces autophagy [40], which regulates replication of intracellular bacteria and is also involved in bacterial antigen presentation in the infected cells.

Several autophagy-related genes have also been reported as CD susceptibility genes. Autophagy is an intracellular process that is involved in degradation and recycling of proteins when cells are in starvation. Autophagy is also involved in the handling of intracellular pathogens. *ATG16L* is a susceptibility gene for CD and is essential for autophagosome formation. Interestingly, it has been shown that NOD1 and NOD2 sense bacterial invasion into the cell and recruit ATG16L to the site of bacterial entry, which triggers autophagy. The intracellular bacteria are then processed through autophagy [40]. This close association between NOD2 and ATG16L suggests the importance of this pathway in the pathophysiology of CD.

Studies on IBD susceptibility genes have revealed the essential role of Paneth cells in CD. Paneth cells reside at the bottom of the intestinal crypts and produce AMPs. The important role of Paneth cells in the regulation of the gut microbiota and the intestinal immune system is shown by the observation that genetically engineered mice overexpressing α-defensin, one of the AMPs, in intestinal epithelial cells had a reduced number of segmented filamentous bacteria (SFB) in the gastrointestinal tract, resulting in impaired Th17 development in the gut [41]. Abnormalities in the size, number, and distribution of granules in Paneth cells, which contain AMPs, have been observed in CD. These morphological abnormalities were reported to be more frequent in CD patients with *NOD2* or *ATG16L* mutations [42]. Mice harboring the same *Atg16l* mutation as CD patients develop similar morphological abnormalities of Paneth cells after murine *Norovirus* infection and become susceptible to dextran sodium sulfate (DSS)-induced colitis [43]. These results provide a good example of the complex interaction between a genetic factor and an environmental factor in the development of intestinal inflammation. Mice deficient in *Xbp1*, which is an essential molecule for endoplasmic reticulum stress and is a CD susceptibility gene, also have impaired autophagy induction in Paneth cells and develop spontaneous ileitis [44]. These results suggest that autophagy in Paneth cells is critical for maintaining gut homeostasis, probably through regulation of the gut microbiota by AMP production. Impaired Paneth cell function may be an essential element in the development and perpetuation of intestinal inflammation in CD.

### How does dysbiosis lead to intestinal inflammation?

It is well known that different commensal bacteria induce distinct types of colitis in IL-10-deficient mice. A mono-association study, in which a single strain of bacteria was inoculated into germ-free IL-10-deficient mice, demonstrated that *E. coli* induced cecal inflammation, *Enterococcus faecalis* induced distal colitis, and *Pseudomonas fluorescens* did not cause colitis [45]. It was also reported that the presence of *Helicobacter hepaticus*, a species of commensal bacteria, exacerbated colitis in IL-10-deficient mice. These results show that alteration of the composition of the gut microbiota can cause distinct intestinal immune responses even in a host with the same genetic background, suggesting that dysbiosis can modulate the immune response in the gut.

Garrett et al. [46] reported that mice deficient in both *Tbx21/T-bet*, which is an essential transcription factor for Th1 differentiation, and *Rag*, which is indispensable for the acquired immune system, developed spontaneous UC-like colitis, which was ameliorated by the administration of antibiotics. Importantly, wild-type mice co-housed with colitic *T-bet/Rag* double knockout mice also developed similar colitis, suggesting that a dysbiotic gut microbiota is communicable and can cause intestinal inflammation without genetic manipulation.

Functional changes in the gut microbiota resulting from dysbiosis may be involved in the pathophysiology of IBD. The number of genes harbored in the gut microbiota is 100 times greater than that in the human genome [5, 47, 48]. Metabolites of the gut microbiota contribute to epithelial cell function, energy balance, and the immune system of the host. A metagenomic analysis of the gut microbiota showed a decrease in genes responsible for carbohydrate and amino acid metabolism and an increase in those in the oxidative stress pathway, in IBD patients [49], raising the possibility that oxidative stress from the gut microbiota causes intestinal inflammation in IBD patients. A specific metabolite of the gut microbiota is also likely to be involved in the pathophysiology of IBD. The gut microbiota metabolizes nonabsorptive dietary fiber and produces short-chain fatty acids such as butyrate and propionate. Commensal bacteria-derived butyrate induces the differentiation of colonic regulatory T cells in mice [50]. Butyrate is also an important energy source for intestinal epithelial cells and increases production of mucin and AMPs [13]. The concentrations of butyrate in feces have consistently been shown to be decreased in IBD patients. Consistently, *F. prausnitzii*, a species of butyrate-producing bacteria, has also been observed to be decreased in IBD [16]. It is possible that the decreased level of butyrate in the gut contributes to inducing intestinal inflammation. Another example of a functional alteration of the gut microbiota in IBD is the increase of sulfate-reducing bacteria (SRB) in UC [51]. SRB produce hydrogen sulfide, which is toxic to the intestinal epithelial cells and can cause mucosal inflammation.

Recent studies have revealed that specific bacteria control the intestinal immune system. SFB, for example, induce Th17 cells in the murine intestine [52]. Although the human counterpart of SFB has not yet been identified, SFB-like organisms were observed in six out of six surgical specimens from UC patients [53]. These SFB-like organisms were not observed in surgical specimens from CD patients. In non-IBD controls, SFB-like organisms were observed in three out of six specimens, with a much lower density compared with UC. These are interesting observations because Th17 cells were reported to be increased in IBD. The physiological role of these SFB-like organisms requires further investigation.

The number of bacteria in the mucus layer is increased in IBD [22], suggesting impaired mucosal barrier function. This is consistent with the fact that many of the UC susceptibility genes are related to mucosal barrier function. Furthermore, bacteria that can degrade mucins in the mucus layer and utilize it as an energy source, for example *Ruminococcus gnavus* and *Ruminococcus torques*, are increased in IBD. These bacteria help other bacteria reside in the mucus layer by providing degraded mucins as nutrients.

## IBD therapies targeting the gut microbiota

### Fecal microbiota transplantation

Fecal microbiota transplantation (FMT) is a treatment to restore abnormal microbial composition of the gut by introducing fecal microbiota obtained from a healthy donor into a diseased individual. The results of a randomized study to compare FMT with antibiotics for recurrent *Clostridium difficile* infection were striking [54]. Resolution of *C. difficile*-associated diarrhea was observed in 13 of 16 patients (81 %) in the FMT group, compared with four out of 13 patients (31 %) in the antibiotics group.

FMT has been highlighted as a treatment to correct dysbiosis in IBD. The first implementation of FMT in UC was reported in 1989 [55]. One of the authors of the paper received FMT for his continuously active UC, resulting in drug-free remission. A recent systematic review identified 18 UC patients without *C. difficile* infection who were treated with FMT [56]. Thirteen out of the 18 patients experienced disease resolution; however, selection bias should be considered because all of the cases were from case reports or small case series.

Two prospective studies of FMT for adult UC patients were recently published [57, 58]. Both of these studies longitudinally analyzed the change of bacterial composition in FMT recipients. Unexpectedly, none of the combined 11 patients in the two studies achieved clinical remission after FMT. In contrast, a significant change in the gut microbiota composition was observed in most of the patients. One paper reported that the successful colonization of donor microbiota was correlated with clinical improvement in one patient, but the other study did not confirm this finding. Both of the papers reported that the alteration of gut microbiota was temporary in most patients, suggesting the necessity of periodically repeated transplantation to maintain the altered gut microbiota.

A phase I trial of FMT for 10 pediatric UC patients with mild-to-moderate activity has recently been completed, reporting no serious adverse events and a high rate of clinical response (79 %) within 1 week [59]. This result is in contrast to the above-mentioned studies. One possible reason to explain this discrepancy is that a certain population of UC patients, but not all, may benefit from FMT. Interestingly, Angelberger et al. identified phylotypes that are indicative of disease severity and FMT success, specifically over-presentation of Enterobacteriaceae and under-repression of Lachnospiraceae [57]. This is an important factor in selecting a subgroup of UC patients that may be responsive to FMT.

### Probiotics

Probiotics are preparations utilizing live bacteria that can be beneficial to human health. Several reports have shown the efficacy of various probiotic bacteria for IBD (Table 2). Efficacy of probiotics was studied more extensively in UC than CD. VSL#3 is a freeze-dried preparation containing eight different lactic acid bacteria (*Lactobacillus acidophilus*, *L. bulgaricus*, *L casei*, *L. plantarum*, *Streptococcus thermophilus*, *Bifidobacterium breve*, *B. infantis*, and *B. longum*). Two double-blinded placebo-controlled trials have shown the efficacy of VSL#3 in the prevention of recurrence in patients with chronic relapsing pouchitis [60, 61]. Two randomized controlled clinical trials showed that addition of VSL#3 to conventional therapy was more efficacious in remission induction in active UC patients [62, 63]. Another randomized controlled trial compared the efficacy of addition of VSL#3 to mesalazine and steroids in 29 newly diagnosed pediatric UC patients with addition of a placebo [64]. The remission rates at 4 weeks were significantly higher in the VSL#3 group (92.8 % (13/14)) than in the placebo group (36.4 % (4/15)). Furthermore, the recurrence rates at 1 year were 36.4 % (3/14) and 73.3 % (11/15) in the VSL#3 and placebo groups, respectively. These results show that VSL#3 is effective for induction of remission as well as its maintenance in UC patients. The mechanism of the anti-inflammatory effect of VSL#3 is not yet fully understood but an increase in regulatory T cells in the gut and upregulation of mucosal alkaline sphingomyelinase have been reported [65].

**Table 2:** Randomized controlled trials of probiotics for inflammatory bowel disease

Probiotics	Disease	Endpoint	Groups and subject no.	Duration	Conclusion	Ref.
VSL#3	UC	Induction	Conventional therapy + VSL#3, 77	12 weeks	Effective	[63]
			Conventional therapy + placebo: 70			
	UC	Induction	Conventional therapy + VSL#3, 71	8 weeks	Effective	[62]
			Conventional therapy + placebo, 73			
	UC	Induction	Steroid/mesalazine + VSL#3, 14	1 year	Effective	[64]
		Maintenance	Steroid/mesalazine + placebo, 15			
	Pouchitis	Maintenance	VSL#3, 20	9 months	Effective	[60]
			Placebo, 20			
	Pouchitis	Maintenance	VSL#3, 20	12 months	Effective	[61]
			Placebo, 16			
*Nissle* 1917	UC	Induction	Steroid + mesalazine, 57	12 weeks	Equivalent to mesalazine	[66]
			Steroid + *Nissle* 1917, 59			
	UC	Maintenance	*Nissle* 1917, 162	12 months	Equivalent to mesalazine	[67]
			Mesalazine, 165			
	UC	Maintenance	Mesalazine, 50	12 weeks	Equivalent to mesalazine	[68]
			*Nissle* 1917, 53			
*Lactobacillus* GG	CD	Maintenance	Conventional therapy + *Lactobacillus* GG, 39	∼2 years	Not effective	[71]
			Conventional therapy + placebo, 36			
	UC	Maintenance	*Lactobacillus* GG, 65	12 months	Equivalent to mesalazine	[70]
			*Lactobacillus* GG + mesalazine, 62			
			Mesalazine, 60			
*Bifidobacteria*-fermented milk (BFM)	UC	Induction	Conventional therapy + BFM, 10	12 weeks	Effective	[74]
			Conventional therapy + placebo, 10			
	UC	Maintenance	Conventional therapy + BFM, 11	12 months	Effective	[75]
			Conventional therapy, 10			
*Bifidobacterium longum*/Synergy 1	UC	Induction	*Bifidobacterium longum*/Synergy 1, 9	1 month	Effective	[73]
			Placebo, 9			

*Nissle 1917*, a nonpathogenic *E. coli* strain, showed efficacy in maintenance of remission in UC equivalent to mesalazine [66–68]. *Nissle 1917* inhibits IL-8 production stimulated by TNFα from the epithelial cells [69]. *Lactobacillus GG* can be effective for maintenance of remission in UC patients [70], but not in CD patients [71, 72]. A pilot study demonstrated that *Bifidobacterium longum/Synergy 1* improved intestinal inflammation in active UC patients [73]. *Bificobacteria*-fermented milk also demonstrated efficacy in induction and maintenance of remission in UC patients [74, 75].

We reported that *Clostridium butyricum* (CB), which is used as a probiotic for patients with functional gastrointestinal disorders in a clinical setting, suppresses intestinal inflammation in murine IBD models [76]. CB potently induced the anti-inflammatory cytokine IL-10 from colonic mucosal macrophages. This IL-10 production was dependent on the Toll-like receptor 2/MyD88 pathway. The effect of CB was abolished in IL-10-deficient mice, suggesting that the anticolitic effect of CB was due to IL-10. Interestingly, heat-killed CB also induced IL-10 from macrophages, strongly indicating that bacterial-derived products are responsible for IL-10 induction. A clinical trial to examine the anticolitic effect of CB in IBD patients is warranted.

## Closing remarks

The gut immune system is separated from an enormous number of bacteria only by a single layer of epithelial cells. It is thus tempting to speculate that the gut microbiota is involved in the pathophysiology of IBD. The advancement of next-generation sequencing technology has revealed a range of alterations of the gut microbiota in IBD. However, it remains unclear whether the dysbiosis observed in IBD is a cause or a consequence of intestinal inflammation. To answer this essential question, studies to examine longitudinal changes of the microbiota in a large number of IBD patients, especially newly diagnosed patients, are necessary. Furthermore, little information is available to how dysbiosis regulates the gut immune system. Understanding the complex relationship between the gut immune system and the microbiota should lead to further elucidation of the pathogenesis of IBD and development of curative treatments. Utilizing the gut microbiota as a diagnostic tool or biomarker is also an attractive idea. To this end, a disease-specific or activity-specific core microbiome should be identified. The gut microbiota is composed not only of bacteria but also of viruses and fungi. It is important to include viruses and fungi in any investigation of the gut microbiota. Furthermore, functional analysis of the gut microbiota in IBD is warranted. Because a majority of the gut bacteria are unable to be cultured, gnotobiotic approaches will be an important tool to investigate the function of the gut microbiota. The gut microbiota represents a “gold mine” for both clinical and basic IBD research.
